# *Ulva pertusa* Modulated Colonic Oxidative Stress Markers and Clinical Parameters: A Potential Adjuvant Therapy to Manage Side Effects During 5-FU Regimen

**DOI:** 10.3390/ijms252312988

**Published:** 2024-12-03

**Authors:** Alberto Repici, Anna Paola Capra, Ahmed Hasan, Rossella Basilotta, Sarah Adriana Scuderi, Michela Campolo, Irene Paterniti, Emanuela Esposito, Alessio Ardizzone

**Affiliations:** 1 Department of Chemical, Biological, Pharmaceutical and Environmental Sciences, University of Messina, 98166 Messina, Italy; alberto.repici@unime.it (A.R.); annapaola.capra@unime.it (A.P.C.); ahmed.hasan@unicam.it (A.H.); rossella.basilotta@unime.it (R.B.); sarahadriana.scuderi@unime.it (S.A.S.); campolom@unime.it (M.C.); ipaterniti@unime.it (I.P.); aleardizzone@unime.it (A.A.); 2 Center of Neuroscience, School of Advanced Studies, University of Camerino, 62032 Camerino, Italy

**Keywords:** fluoropyrimidines, 5-Fluorouracil (5-FU), side effects, *Ulva pertusa*, anti-inflammatory and antioxidant markers, clinical biochemistry parameters

## Abstract

One of the most used chemotherapy agents in clinical practice is 5-Fluorouracil (5-FU), a fluorinated pyrimidine in the category of antimetabolite agents. 5-FU is used to treat a variety of cancers, including colon, breast, pancreatic, and stomach cancers, and its efficacy lies in its direct impact on the patient’s DNA and RNA. Specifically, its mechanism blocks the enzymes thymidylate synthetase and uracil phosphatase, inhibiting the synthesis of uracil, which cannot be incorporated into nuclear and cytoplasmic RNA. Despite being one of the most used drugs in oncology, it is associated with several significant side effects, including inflammation of the mouth, loss of appetite, and reduction in blood cells. In our study, we examined the reduction of side effects in a 5-FU regimen administered at doses of 15 mg/kg and 6 mg/kg for 14 days in 6-week-old male Sprague-Dawley rats. On the 14th day, the rats were treated orally for 2 weeks with 100 mg/kg of *Ulva pertusa*, a well-known seaweed from the Ulvaceae family, which has demonstrated powerful biological properties. The administration of this green alga alleviated the side effects of 5-FU, improving several parameters including body weight, food intake, and diarrhea index. It also helped reduce side effects in the blood, kidneys, and liver. Histological and molecular analyses were conducted on serum and colon tissues from the rats, examining changes in colon structure and the release of oxidative stress markers such as iNOS, COX-2, and nitrotyrosine. Several biochemical indicators, including SOD, CAT, GSH, MDA, and ascorbic acid, were also evaluated. Overall, our data indicated *Ulva pertusa* to be a promising therapeutic against 5-FU’s adverse effects, therefore, it could be worthwhile to investigate the possibility of using this alga in safer cancer treatment formulations. Certainly, future preclinical and clinical studies could assess the alga’s efficacy in diverse cancer treatment regimens, exploring its role as an adjuvant therapy that may reduce chemotherapy-related toxicity without compromising therapeutic outcomes.

## 1. Introduction

Chemotherapy is a crucial cancer treatment, as it targets and destroys rapidly dividing cancer cells throughout the body and it can be effective in shrinking tumors, reducing symptoms, and preventing the spread of cancer to other areas [1]. Chemotherapy is often used in combination with other treatments, such as surgery and radiation, to increase the chances of remission or cure [2], and it certainly plays a vital role in managing cancers that are inoperable or have metastasized, offering patients a chance at prolonged survival and improved quality of life [2].

In this wide range of drugs, 5-FU is a chemotherapeutic agent classified as an antimetabolite, which interferes with the synthesis of DNA and RNA, thereby preventing cancer cells from growing and dividing [3].

Once administered, 5-FU is converted within cells into several active metabolites. One key metabolite, 5-fluoro-2′-deoxyuridine monophosphate (FdUMP), binds to and irreversibly inhibits the enzyme thymidylate synthase, which is essential for the synthesis of thymidylate, a crucial DNA component [4]. Without thymidylate, DNA replication is halted, leading to irreparable damage to cancer cells [4]; additionally, other 5-FU metabolites can be directly incorporated into RNA, disrupting protein synthesis and further contributing to cell death [4].

This drug is particularly effective against various types of solid tumors, such as those in the colon, rectum, breast, and head and neck [5], and because of its targeted action, 5-FU has become a cornerstone in the fight against cancer, often used in monotherapy or in combination with other drugs to enhance therapeutic outcomes [5].

Despite its efficacy, 5-FU can cause a range of adverse reactions, which can be influenced by both genetic and non-genetic factors [6].

On a genetic level, individuals with dihydropyrimidine dehydrogenase (DPD) deficiency are at a significantly higher risk of severe toxicity [7], since DPD is an enzyme responsible for breaking down 5-FU in the body [7].

An enzymatic deficiency, often due to genetic mutations, results in the accumulation of toxic levels of the drug, leading to potentially life-threatening side effects such as severe myelosuppression, neurotoxicity, and even death [7]. Therefore, genetic screening for DPD deficiency before starting 5-FU therapy can be crucial for preventing these severe adverse reactions [7].

Other side effects include gastrointestinal symptoms like nausea, vomiting, and diarrhea, as well as myelosuppression, leading to anemia, leukopenia, and thrombocytopenia [8]. Moreover, patients may also experience mucositis, hand-foot syndrome, and skin rashes [8].

Patients undergoing treatment with 5-FU often experience an increase in oxidative stress, which can significantly impact their overall health and treatment outcomes [9]. 5-FU induces the generation of reactive oxygen species (ROS) within cells, leading to oxidative damage to lipids, proteins, and DNA [10]. This heightened oxidative stress contributes to both the therapeutic effects and the side effects of 5-FU, as it enhances cancer cell death but also damages healthy tissues.

The imbalance between ROS production and the body’s antioxidant defenses can exacerbate side effects such as cardiotoxicity, mucositis, and hand-foot syndrome [11]. In some cases, elevated oxidative stress may even lead to chronic inflammation and further complications during treatment [11]. As a result, managing oxidative stress through antioxidants or other supportive therapies is being explored to potentially improve the tolerability of 5-FU in cancer patients.

In recent years, there has been a significant increase in the use of natural compounds to reduce oxidative stress, which plays a central role in the development of various human chronic diseases [12].

This growing interest in natural antioxidants extends to their potential use in mitigating the adverse effects of chemotherapy [13]. Therefore, the incorporation of these natural antioxidants into cancer treatment regimens holds promise for improving patient outcomes by decreasing the harmful side effects of conventional chemotherapy.

Here, we focused on the green alga *Ulva pertusa*, a species widely recognized as a valuable food source across various regions worldwide, largely due to its rich content of dietary fiber, bioactive polysaccharides, proteins, lipids, essential vitamins (such as A, C, E, and B-complex), and critical minerals, including calcium, iron, and magnesium [14]. This diverse nutrient profile not only makes *Ulva pertusa* a highly nutritious component of the diet but also contributes to its extensive use in various therapeutic applications.

Owing to its antihyperlipidemic, immunomodulatory, and notably potent antioxidant activities, *Ulva pertusa* has gained considerable interest within both traditional and modern medicine [15]. In traditional Chinese medicine, it has been utilized for centuries to help manage and alleviate a variety of conditions, ranging from digestive disorders and liver health issues to inflammatory diseases and infections [15].

The antioxidant properties of *Ulva pertusa* are especially noteworthy, as its components can neutralize free radicals, reducing oxidative stress at the cellular level [16]. Additionally, the immunomodulatory effects of *Ulva pertusa* make it a compelling candidate for further exploration in immune support and regulation, potentially enhancing the body’s defenses against infections while also tempering excessive immune responses that lead to inflammation and autoimmunity [17].

In our extract, these beneficial properties can be attributable to the active ingredients present in this alga, and in particular to the polysaccharide fraction which increases the anti-inflammatory activity, to the amino acid fraction capable of modulating the intestinal microbiota, and to the presence of polyunsaturated fatty acids (PUFA), ω-3 and ω-6 having powerful antioxidant activity [17,18].

Together, these properties position *Ulva pertusa* as an attractive candidate for the development of functional foods and nutraceuticals aimed at enhancing overall health and well-being. Its application in pharmaceuticals, as well as in health supplements, is a growing area of research, given the alga’s potential to serve as a natural source of bioactive compounds that support health on multiple levels. As research continues to reveal more about its therapeutic benefits, *Ulva pertusa* holds considerable promise for inclusion in dietary interventions and therapeutic regimens, particularly those targeting oxidative stress, immune modulation, and metabolic health.

In our previous studies, we already demonstrated the anti-inflammatory, antioxidant, immunomodulatory, and analgesic activities of *Ulva pertusa* in the colonic environment using a murine model of colitis [17,18].

Therefore, building on those promising results, the aim of this study was to further explore the potential beneficial effects of *Ulva pertusa* extract in other pathological contexts, specifically within a chemotherapeutic regimen, using a rodent model of 5-FU-induced side effects.

Overall, the present paper could have a significant impact on the oncology field by improving the quality of life of patients undergoing chemotherapy such as 5-FU, and introducing innovative cancer treatment strategies that exploit the benefits of natural compounds, suggesting their potential as a general additional therapy.

## 2. Results

### 2.1. Ulva pertusa Decreased Weight Loss, Diarrhea and Increased Food Intake During the 5-FU Regimen

**Figure 1:**
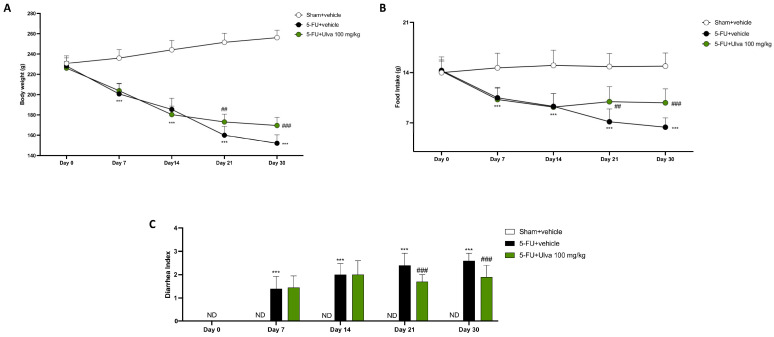
Effect of the green alga *Ulva pertusa* on body weight loss, food intake, and diarrhea index. Graphical representation of body weight (**A**), food intake (**B**), and diarrhea index (**C**). The number of rats in every experimental group was eight. Data are expressed as means ± SD. Two-way ANOVA test. ND: Not Detectable; *** *p* < 0.001 vs. Sham; ## *p* < 0.01 vs. 5-FU + vehicle; ### *p* < 0.001 vs. 5-FU + vehicle.

We used different methods to assess body weight, food intake, and diarrhea to better understand the potential beneficial effects of *Ulva pertusa* against these side effects caused by 5-FU. Rats injected with 5-FU showed significantly worse results in all trials in a time-dependent way compared to Sham (Figure 1A–C); notably, oral administrations of *Ulva pertusa* effectively lessened the three typical adverse effects that occur after receiving 5-FU chemotherapy. Indeed, comparing the treatment group with the 5-FU + vehicle group, the results showed the beneficial impact of *Ulva pertusa* 100 mg/kg in reducing weight loss (Figure 1A) and diarrhea (Figure 1C) as well as in increasing food intake (Figure 1B). In particular, improvements in all of these parameters were observed after day 21 (1 week of *Ulva pertusa* administration) and increased until day 30 (end of the experiment).

### 2.2. Ulva pertusa Reduced Pain Sensitivity and Photophobia Caused by 5-FU Injections

Increased sensitivity to pain and light may be debilitating side effects for patients undergoing 5-FU therapy. Therefore, we employed the light/dark and the Von Frey tests to assess photophobia and hyperalgesia, respectively.

**Figure 2:**
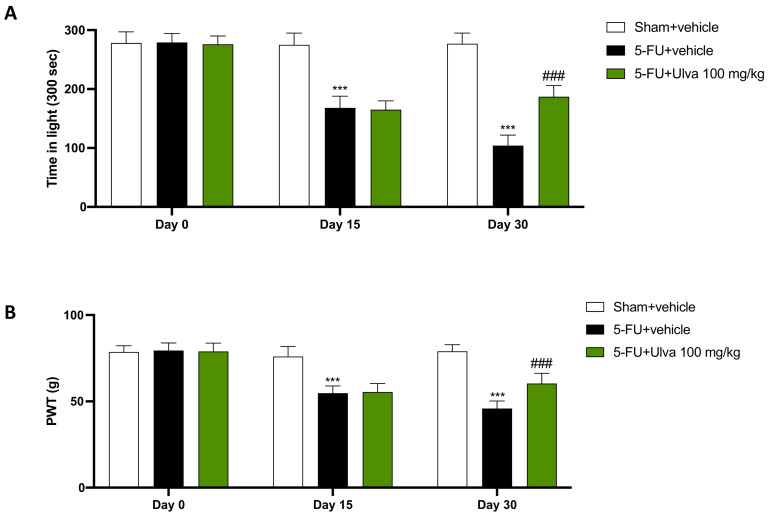
Positive modulation of photophobia and pain sensitivity by *Ulva pertusa*. Behavioral analysis of the rats such as light/dark test (**A**) and von Frey test (**B**) at time 0, after 15 days and 30 days. The number of rats in every experimental group was eight. Data are expressed as means ± SD. One-way ANOVA test. *** *p* < 0.001 vs. Sham; ### *p* < 0.001 vs. 5-FU + vehicle.

In the light/dark test, 5-FU + vehicle rats had significantly higher photophobia than Sham animals (Figure 2A), while oral treatment with *Ulva pertusa* greatly improved the light tolerance of animals, allowing 5-FU-treated animals to be kept in the illuminated area for longer periods (Figure 2A).

Likewise, the Von Frey test measures pain sensitivity in rats by applying mechanical stimuli with filaments of different stiffness on the paws, evaluating the PWT, which indicate the threshold for a painful response. Sham rats showed a better pain threshold than 5-FU + vehicle rats, which exhibited significantly lower threshold values (Figure 2B); however, rats treated with *Ulva pertusa* 100 mg/kg had reduced pain sensitivity compared to vehicle animals (Figure 2B).

### 2.3. Ulva pertusa Supplementation Reduced Tissue Damage and Neutrophil Infiltration in Colon Tissues Following 5-FU Regimen

One major and incapacitating adverse effect of 5-FU is colon damage, which can seriously impair a patient’s quality of life and daily activities. In light of this, we employed the H&E stain to check colon morphology following the 5-FU regimen.

**Figure 3:**
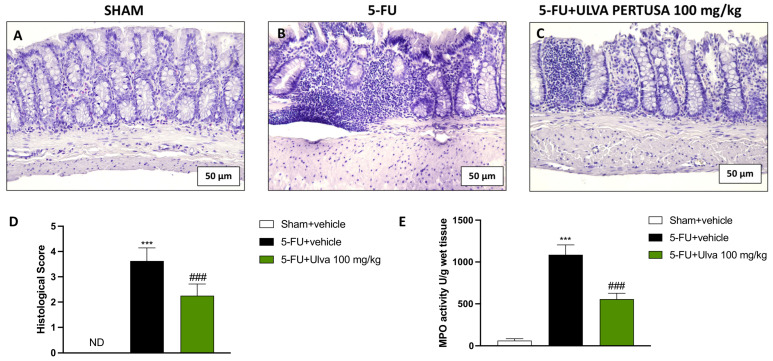
Evaluation of colon damage and neutrophil infiltration. H&E representatives’ images of colon from Sham (**A**,**D**), 5-FU + vehicle (**B**,**D**), and 5-FU + Ulva pertusa 100 mg/kg (**C**,**D**). Neutrophilic activity was assessed by MPO assay (**E**). The number of rats in every experimental group was eight. The histological evaluations are represented at 20× magnification. Data are expressed as means ± SD. One-way ANOVA test. ND: Not detectable; *** *p* < 0.001 vs. Sham; ### *p* < 0.001 vs. 5-FU + vehicle.

The H&E staining confirmed that the colon tissues from 5-FU animals (Figure 3B,D) were severely damaged when compared to Sham animals’ samples (Figure 3A,D). Treatment with *Ulva pertusa* 100 mg/kg (Figure 3C,D) restored the morphological architecture of rat colons to a remarkable degree.

A fast way to track the neutrophil activity is the MPO assay, as the activity of the enzyme myeloperoxidase is considered as a benchmark for inflammation. Neutrophil infiltration was also abundant in animals of the 5-FU + vehicle group compared to Sham animals (Figure 3E). Nevertheless, neutrophils were meaningfully reduced by oral treatment with *Ulva pertusa* 100 mg/kg (Figure 3E).

### 2.4. Ulva pertusa Administration Reduced COX-2 Expression and Lipid Peroxidation in Colon Tissues Following 5-FU Regimen

A general state of inflammation is found in the colon during 5-FU intake. By using immunohistochemistry, we assessed the local expression of COX-2 as a well-known inflammatory marker in rat bowel tissues after 5-FU intoxication.

**Figure 4:**
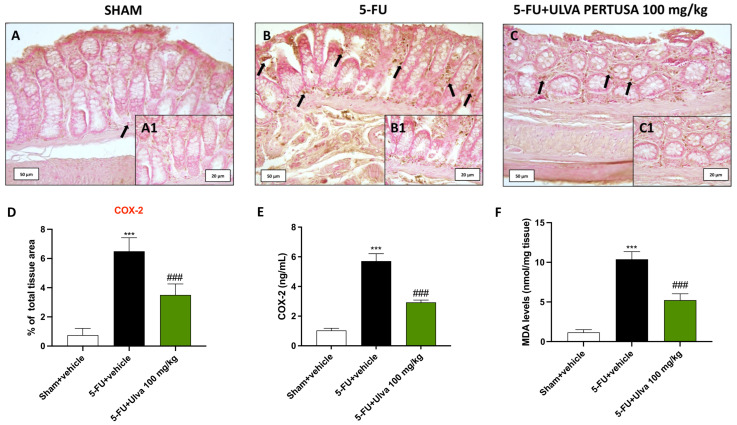
Evaluation of COX-2 expression and MDA levels in colon tissues. Immunohistochemical staining of COX-2 in colon tissue from Sham (**A**,**A1**), 5-FU + vehicle (**B**,**B1**), and 5-FU + *Ulva pertusa* 100 mg/kg (**C**,**C1**). Immunohistochemical score (**D**). ELISA kit for COX-2 (**E**). MDA assay (**F**). The number of rats in every experimental group was eight. Black arrows indicate immunopositivity for COX-2 in the colonic cells. The pictures are displayed at 20× and 40× magnification. Data are expressed as means ± SD. One-way ANOVA test. *** *p* < 0.001 vs. Sham; ### *p* < 0.001 vs. 5-FU + vehicle.

Our results showed that rats from the 5-FU + vehicle group had higher intestinal levels of COX-2 (Figure 4B,B1,D), compared to Sham tissues (Figure 4A,A1,D). However, the percentage of COX-2 immunopositive cells was significantly reduced by oral administration of *Ulva pertusa* 100 mg/kg (Figure 4C,C1,D).

Immunohistochemical findings were confirmed by ELISA kit quantification (Figure 4E).

Furthermore, the MDA assay, as a biomarker for lipid peroxidation, was also performed to confirm the inflammatory status, also allowing oxidative stress and cell damage to be assessed.

From the data obtained, rats in the group 5-FU + vehicle had significantly higher colonic MDA levels compared to basal parameters from Sham animals (Figure 4F). Treatment with *Ulva pertusa* 100 mg/kg effectively reduced the lipidic peroxidation as shown by the decreased MDA levels compared to 5-FU + vehicle animals (Figure 4F).

### 2.5. Ulva pertusa Administration Mitigated iNOS Immunopositivity in Colon Tissues Following 5-FU Regimen

iNOS plays a vital role in the pattern of inflammation, hence, its expression was examined in the colon tissues of rats using immunohistochemistry.

**Figure 5:**
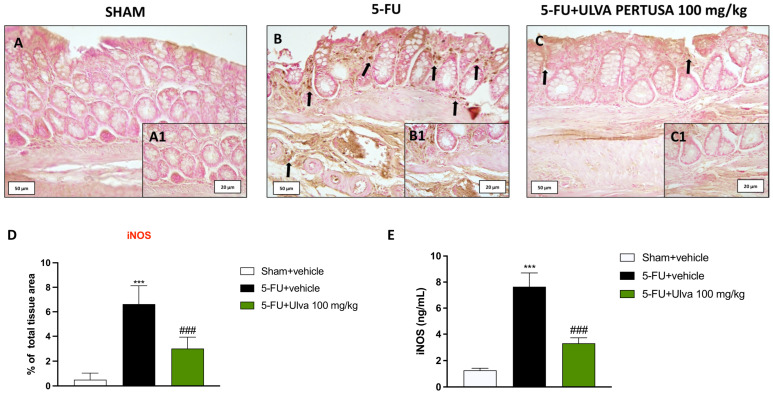
Evaluation of iNOS expression in colon tissues. IHC staining showed the trend of iNOS expression in rat colon tissues from Sham (**A**,**A1**), 5-FU + vehicle (**B**,**B1**), and 5-FU + *Ulva pertusa* 100 mg/kg (**C**,**C1**). Immunohistochemical score (**D**). ELISA kit for iNOS (**E**). The number of rats in every experimental group was eight. Black arrows indicate immunopositivity for COX-2 in the colonic cells. The pictures are presented at 20× and 40× magnification. Data are expressed as means ± SD. One-way ANOVA test. *** *p* < 0.001 vs. Sham; ### *p* < 0.001 vs. 5-FU + vehicle.

Compared to Sham animals (Figure 5A,A1,D), animals subjected to a 5-FU + vehicle regimen presented greater levels of iNOS expression in colon tissues (Figure 5B,B1,D).

On the other hand, when *Ulva pertusa* 100 mg/kg was orally administered, the levels of iNOS in the colons were considerably decreased (Figure 5C,C1,D).

The immunohistochemical results were validated by protein quantification using the ELISA kit (Figure 5E).

### 2.6. Ulva pertusa Administration Reduced Nitrotyrosine Expression in Colon Tissues Following 5-FU Regimen

Nitrotyrosine is a biomarker that can be applied to monitor the nitrosative damage triggered by 5-FU medical therapy. Therefore, we performed immunohistochemical localization for this marker in colon samples.

**Figure 6:**
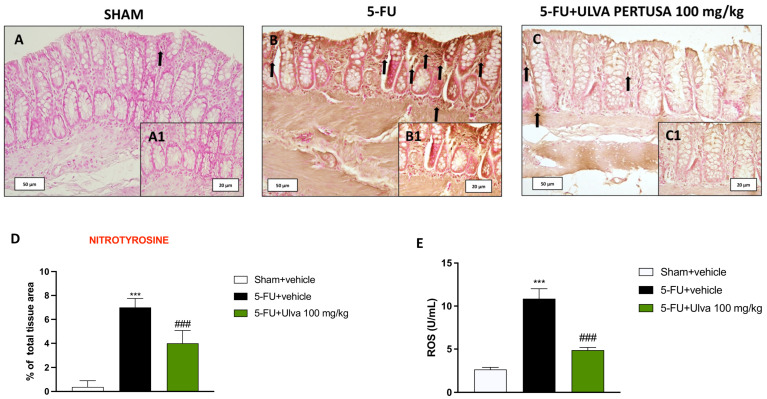
Immunolocalization of nitrotyrosine in colon tissues. Immunohistochemical staining for nitrotyrosine in the colon tissues from Sham group (**A**,**A1**), 5-FU + vehicle group (**B**,**B1**), and 5-FU + *Ulva pertusa* 100 mg/kg group (**C**,**C1**). Immunohistochemical score (**D**). ELISA kit for ROS quantification (**E**). The number of rats in every experimental group was eight. Black arrows indicate immunopositivity for COX-2 in the colonic cells. The pictures are presented at 20× and 40× magnification. Data are expressed as means ± SD. One-way ANOVA test. *** *p* < 0.001 vs. Sham; ### *p* < 0.001 vs. 5-FU + vehicle.

From our data, nitrotyrosine expression was notably higher in the colon tissues of the 5-FU + vehicle group (Figure 6B,B1,D) compared to the Sham animals (Figure 6A,A1,D). Daily oral treatment with *Ulva pertusa* 100 mg/kg significantly reduced nitrotyrosine expression (Figure 6C,C1,D).

Moreover, considering the importance of oxidative stress in spreading colon damage, we assessed ROS levels by immunosorbent assay.

The results obtained for ROS levels showed significant differences between the Sham and 5-FU groups. Indeed, in the 5-FU-treated group, there was a marked increase in ROS production in colon samples compared to the Sham group (Figure 6E).

Treatment with *Ulva pertusa* resulted in a significant reduction in ROS levels compared to the vehicle group, indicating a protective effect in the colonic environment against oxidative stress induced by 5-FU (Figure 6E).

### 2.7. Effects of Ulva pertusa Administration on Blood Cells During 5-FU Regimen

Pancytopenia is one of the most frequent adverse reactions to chemotherapy. Therefore, on days 15 and 30, we extracted blood from the animals to assess any potential positive effects of *Ulva pertusa*. This allowed us to investigate the impact of *Ulva pertusa* on the blood cells negatively impacted by 5-FU. Blood cells were assessed twice during the experiment: once on day 15, before starting *Ulva pertusa* treatment, and again on day 30, at the end of the experiment.

**Figure 7:**
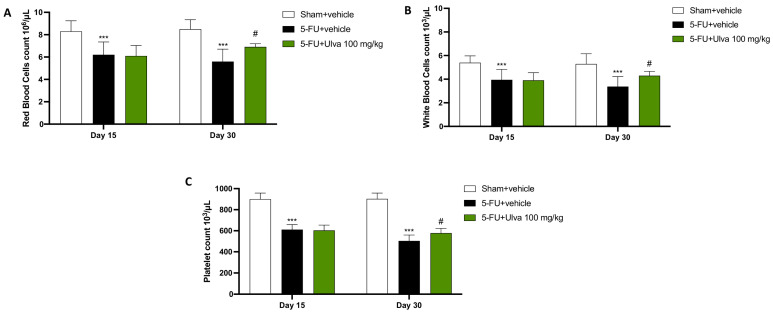
Blood cells count. The red (**A**) and white (**B**) blood cells and platelets (**C**) were evaluated on day 15 and 30 by using an automated cell counter. The number of rats in every experimental group was eight. Data are expressed as means ± SD. One-way ANOVA test. *** *p* < 0.001 vs. Sham; # *p* < 0.05 vs. 5-FU + vehicle.

In this experiment, rats from the 5-FU + vehicle group had considerably fewer red blood cells (Figure 7A), white blood cells (Figure 7B), and platelets (Figure 7C) on day 15 than the Sham group.

All parameters were partially restored, showing an appreciable recovery, after 2 weeks of treatment with *Ulva pertusa* 100 mg/kg (Figure 7A–C).

### 2.8. Therapeutic Potential of Ulva pertusa in Renal and Liver Function Support

**Figure 8:**
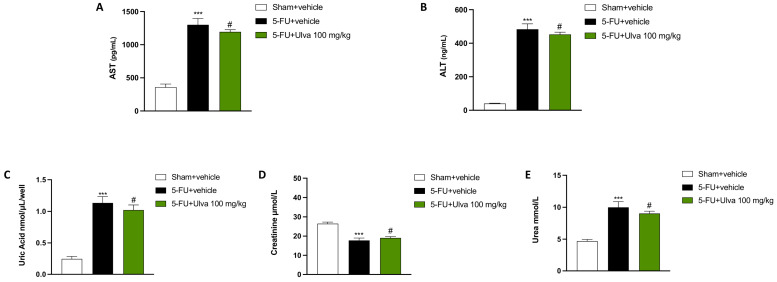
Renal and hepatic evaluations. Using ELISA kits, AST (**A**), ALT (**B**), uric Acid (**C**), creatinine (**D**) and urea (**E**) were evaluated. The number of rats in every experimental group was eight. The results are expressed as means ± SD. One-way ANOVA test. *** *p* < 0.001 vs. Sham; # *p* < 0.05 vs. 5-FU + vehicle.

Renal and hepatic failure is one of the most common side effects during chemotherapy with 5-FU. Liver dysfunction was evident in 5-FU + vehicle-treated rats (Figure 8A,B) compared to the Sham group (Figure 8A,B), with a noticeable increase in AST and ALT levels.

*Ulva pertusa* 100 mg/kg administration partially reduced the liver damage induced by 5-FU, confirming an appreciable capacity to manage the toxic effects of this chemotherapeutic agent (Figure 8A,B).

The most classic parameters of renal clearance are uric acid, creatinine, and urea, which allow a full analysis of kidney function and health status.

In all the kits conducted, rats injected with 5-FU + vehicle (Figure 8C–E) showed significantly altered renal parameters compared to the physiological levels of the Sham group (Figure 8C–E), indicating significant renal damage.

Oral treatment with *Ulva pertusa* 100 mg/kg (Figure 8C–E) slightly mitigated kidney damage, suggesting a moderate efficacy in protecting against the 5-FU-induced renal toxicity.

### 2.9. Ulva pertusa Administration Enhanced Endogenous Antioxidant Defense Previously Impaired by 5-FU Chemotherapy

5-FU exerts a strong and disseminated effect on the body of patients, and this action often extends to the endogenous antioxidant defenses. Hence, at the end of the experiment, we employed ELISA kits to assess the levels of GSH, CAT, SOD1, and vitamin C.

**Figure 9:**
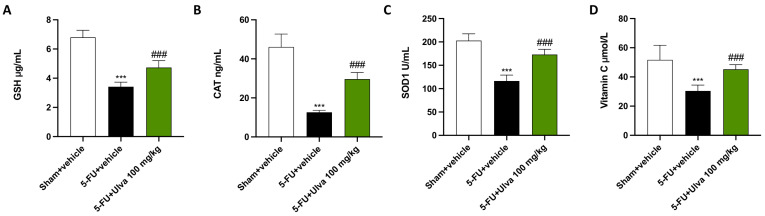
Biochemical analysis of antioxidant defense markers. Serum analysis of GSH (**A**), CAT (**B**), SOD (**C**) and Vitamin C (**D**). The number of rats in every experimental group was eight. Data are expressed as means ± SD. One-way ANOVA test. *** *p* < 0.001 vs. Sham; ### *p* < 0.001 vs. 5-FU + vehicle.

In every test performed, we observed a severe reduction of all parameters in the 5-FU + vehicle group compared to the Sham group (Figure 9A–D).

However, *Ulva pertusa* 100 mg/kg supplementation led to a considerable improvement in GSH, CAT, SOD, and vitamin C contents (Figure 9A–D).

## 3. Discussion

Managing chemotherapy’s side effects during treatment remains a critical aspect of adjuvant therapies [19]. The legitimacy of an anticancer treatment depends not only on its ability to fight cancer but also on its effect on the patient’s quality of life.

5-FU’s mechanism of action, which includes inhibiting thymidylate synthase, an enzyme essential to DNA synthesis and repair, explains its therapeutic success. 5-FU inhibits this enzyme, which prevents thymidine from being synthesized, a nucleotide that is necessary for DNA replication [20]. Instead of uridine triphosphate (UTP), this antimetabolite is integrated into RNA, disrupting its processing and function and preventing the synthesis of proteins [20]. Because of the overabundance of growth factors or the absence of inhibitory proteins, this disruption causes DNA damage and cell death, which is especially harmful to cancer cells which divide quickly [21].

Previously, Ferguson and colleagues investigated the protective benefits of curcumin in vitro, which were facilitated by 5-FU’s ability to protect healthy cells, as well as the possibility of greater dosages and longer treatment durations. This research attempted to minimize adverse effects, also highlighting the potential benefits deriving from natural compounds during chemotherapeutic regimens [22]. In addition, in vitro research on propolis extract as a potential adjuvant for 5-FU antitumor treatment confirmed its selective impact on cancer cells and not on healthy ones [23].

In line with this, here, we aimed to examine the likely relieving effects of *Ulva pertusa* on 5-FU side effects, with a special focus on the gut environment and inflammatory or oxidative stress markers.

5-FU therapy for cancer patients is linked to several adverse effects, such as inappetence and a notable loss of body weight [24]. These symptoms are especially concerning, since consuming fewer calories and losing body mass as a result might impair responsiveness to therapy, lower quality of life, and raise the risk of complications. 5-FU has a detrimental effect on the mucosa of the gastrointestinal tract, leading to nausea, vomiting, and inflammation. These side effects exacerbate nutritional status and cause appetite loss [24]. In this study, *Ulva pertusa* oral therapy improved physical conditions, specifically body weight and the nutritional status of rats.

Diarrhea is a common and debilitating side effect of 5-FU chemotherapy. This side effect can significantly impair the daily activities of patients and limit the dose of chemotherapy that can be administered. Several varieties of seaweed appear to have similar anti-diarrheal properties [25], for instance, species such as *Gracilaria* [26] and *Laminaria japonica* [27] have shown comparable benefits in reducing intestinal motility and improving colonic water absorption, both of which aid in the relief of diarrhea. In addition, seaweed’s high dietary fiber content can encourage the development of good gut flora, enhancing digestive health and lessening the frequency and intensity of diarrhea. *Ulva pertusa*, at a dose of 100 mg/kg, has shown effective anti-diarrheal properties, significantly reduced diarrhea and demonstrated effective beneficial effects after 14 days of therapy.

Treatment with 5-FU may induce two significant side effects: photophobia and hyperalgesia. Photophobia is a condition characterized by excessive sensitivity to light. In patients treated with chemotherapy, this may occur due to alterations in the nervous system that affect normal visual perception [28], making it uncomfortable or painful to be exposed even to light of normal intensity. This symptom is often related to drug-induced neurotoxicity, which can affect the optic nerves or other areas of the sensory system [29].

On the other hand, hyperalgesia is characterized by an increased susceptibility to pain, making even minor stimuli uncomfortable. In chemotherapeutic-treated patients, hyperalgesia may result from aberrant nociceptive pathway activation, which transmits pain signals [30]. Due to the drug’s ability to increase inflammation and change neurotransmitter function, patients may experience extreme pain even in reaction to insignificant or painless stimuli [30].

In this context, our results showed that *Ulva pertusa* therapy improved both photophobic condition and pain sensitivity, demonstrating it to be a good adjuvant in reducing 5-FU treatment-related discomfort.

Chemotherapy-induced colitis is a significant gastrointestinal side effect that can occur with 5-FU treatment. Acute colon inflammation, which is the defining feature of this illness, is frequently accompanied by severe diarrhea, gastrointestinal pain, and, in more severe instances, rectal bleeding [31]. 5-FU causes damage to the intestinal epithelial cells, which changes the mucosal barrier, causing inflammation, and increasing the possibility of bacterial infections recurring. Because 5-FU causes inflammation along with a compromised immune system, colitis can result in consequences like neutropenic colitis (or neutropenic enterocolitis), which can be deadly if left untreated [32].

Here, according to the histological examinations, the oral administration of *Ulva pertusa* has been advantageous in improving colon morphology as well as decreasing the amount of inflammatory cells that infiltrate the colon’s mucosa and submucosal layer such as neutrophils.

The inflammatory state produced by chemotherapy such as 5-FU represents a serious side effect of therapy; inflammation can even result in therapeutic failure [33]. In this regard, COX-2 is one of the most important pro-inflammatory markers that can be evaluated, playing a key role in the synthesis of prostaglandins and the modulation of the inflammatory process [34]. iNOS is responsible for the production of nitric oxide (NO), is mainly expressed in pathological conditions, and contributes to the inflammatory response and defense against pathogens [35]. Nitrotyrosine is formed in the presence of the radical NO through the pathway that leads to the oxidation of peroxynitrite, so its determination reflects endothelial damage due to the radical NO.

Our data demonstrated *Ulva pertusa*’s anti-inflammatory properties in the colon, as indicated by the reduced expression of COX-2, iNOS, and nitrotyrosine. Moreover, the remarkable anti-inflammatory and antioxidant properties of *Ulva pertusa* have also been valuable in decreasing lipidic peroxidation and ROS levels in the colon.

Myelosuppression is one of the most serious side effects associated with chemotherapy treatment such as 5-FU [36]. The process of hematopoiesis, which takes place in the bone marrow, produces blood cells. This process begins with progenitor stem cells, which have the ability to differentiate into different hematopoietic lines that give rise to white blood cells, red blood cells, and platelets [37]. The drugs used for chemotherapy destroy these progenitor cells because, although they are successful in eradicating cancer cells, they also frequently affect bone marrow cells that divide quickly, which leads to myelosuppression [38].

Thus, this potentially fatal and severely debilitating effect has been examined in this study in two different timeframes. We found that after 15 days of chemotherapeutic induction, the animals presented a high degree of leukopenia, anemia, and thrombocytopenia, which developed simultaneously as a direct result of 5-FU’s activity. On the other hand, data demonstrated that prolonged treatment for 2 weeks with *Ulva pertusa*, a seaweed rich in vitamins, minerals, and essential amino acids crucial for the production and maturation of blood cells, effectively increased the number of blood cells.

5-FU may cause hepatocellular damage in patients by raising blood levels of hepatic enzymes including ALT and AST, which are indicators of liver toxicity. 5-FU damages the mitochondria of the hepatocytes and induces oxidative stress in the liver, which leads to inflammation [39]. In the most extreme situations, this can cause toxic hepatitis in patients, which is characterized by exhaustion, abdominal pain, and jaundice. Hepatic toxicity from 5-FU can sometimes progress to liver fibrosis or steatosis, which would impair liver function overall [40].

Therefore, an imbalance in these markers suggests hepatotoxicity or liver damage, which is widespread in individuals after 5-FU treatment. From our data, *Ulva pertusa* treatment appreciably decreased the AST and ALT levels, also indicating a possible hepatoprotective benefit due to its biological properties.

The kidneys are also often damaged by the action of 5-FU, as its metabolites induce oxidative stress, inflammation, and direct cytotoxicity in renal cells, leading to conditions such as acute kidney injury (AKI) or chronic nephrotoxicity, which can significantly impair renal function and exacerbate the overall toxicity of chemotherapy. In this context, uric acid as the final product of purine metabolism, creatinine as a waste product of muscle metabolism, and urea as one of the waste products of protein metabolism, represent three trusty indicators of renal health [41].

From our assessments, *Ulva pertusa* significantly modulated the levels of uric acid, creatinine, and urea, demonstrating a protective effect on kidney function. These findings suggest that *Ulva pertusa* may also play a beneficial role in preserving renal health by reducing the metabolic burden and preventing kidney damage.

Chemotherapy treatment can significantly impair the body’s natural antioxidant defenses, leading to an imbalance between the generation of free radicals and the body’s capacity to neutralize them. 5-FU may decrease the activity of important antioxidant enzymes such as GSH, SOD, CAT, and Vitamin C by raising oxidative stress at the cellular level [42]. This deterioration of antioxidant defenses exacerbates tissue toxicity, particularly in organs like the liver and kidneys, by causing oxidative damage to proteins, lipids, and DNA.

However, in the present study, rats orally administered with *Ulva pertusa* demonstrated considerably higher levels of antioxidant markers such as GSH, CAT, SOD, and vitamin C, highlighting an overall strengthening of the endogenous antioxidant defense.

Therefore, based on these results, the integration of natural compounds with chemotherapy could be considered a new strategy that might lead to better therapeutic results, bridging a significant gap in current cancer treatment paradigms.

Focusing on reducing side effects, this research directly addresses a critical patient concern, making the results highly relevant and applicable in clinical settings.

Of interest, in line with our findings, several in vivo studies have highlighted the potential of natural compounds to mitigate the toxicity associated with 5-FU, offering points of comparison with the protective effects of *Ulva pertusa*.

Thymol, a bioactive compound derived from thyme, has demonstrated hepatoprotective effects by modulating oxidative stress and apoptotic signaling pathways, including Akt/GSK-3β and MAPK (ERK1/2). By targeting these pathways, thymol reduces liver damage caused by 5-FU and prevents cellular apoptosis through its potent antioxidant and anti-inflammatory properties [43].

Thymoquinone (TQ), a compound extracted from *Nigella sativa*, has been explored for its role in reducing 5-FU-induced toxicity, particularly when incorporated into advanced drug delivery systems such as calcium carbonate nanoparticles. This combination enhances the therapeutic efficacy of 5-FU by improving its bioavailability and providing targeted, sustained release. Notably, TQ also exhibits anti-inflammatory and antioxidant properties, which synergistically reduce the adverse effects of chemotherapy [44].

Other studies have investigated the benefits of polyphenol- and flavonoid-rich plant extracts, which counteract 5-FU-induced damage by scavenging ROS and reducing inflammatory responses. These compounds, derived from sources such as green tea and other medicinal plants, have shown efficacy in protecting against systemic and gastrointestinal toxicity [45].

However, despite these encouraging results, we must consider the limitations of this study. Interactions between natural compounds and chemotherapy involve complex biological mechanisms that are not yet fully understood, making their clinical application difficult. Although pre-clinical models offer valuable information, they often fail to take into account the physiological and genetic diversity present in human patients. As a result, translating these results into effective treatments requires additional validation through clinical trials.

## 4. Materials and Methods

### 4.1. Materials

The chemicals utilized in this study were of the finest commercial grade available. Unless specified otherwise, all compounds were sourced from Sigma-Aldrich (Milan, Italy). Stock solutions were prepared using non-pyrogenic saline (0.9% NaCl; Baxter, Liverpool, UK). The *Ulva pertusa* extract was kindly provided by the ChiBioFarAm Department at the University of Messina (Messina, Italy).

The *Ulva pertusa* extract was prepared using a standardized methodology to ensure the consistency and integrity of the bioactive compounds, as we did previously for other marine species [46]. After alga harvesting, it was thoroughly washed to remove debris and contaminants, and dried under controlled conditions to prevent degradation. After homogenization and lyophilization, the dried material was then ground into a fine powder and subjected to extraction using a specific solvent optimized for isolating target bioactive compounds. The solvents used in extractions and sample preparation were of high-performance liquid chromatography (HPLC; Agilent Technologies, Santa Clara, CA, USA) or liquid chromatography coupled with mass spectroscopy (LC/MS; Agilent Technologies, Santa Clara, CA, USA) grade, and were purchased from Sigma-Aldrich (Milan, Italy) [46].

Then, the extract was stored under optimal and controlled conditions to ensure its chemical stability and bioactive properties. Specifically, the extract, prepared as a dry powder, was stored at controlled humidity and temperature, in airtight containers protected from light and equipped with desiccants to prevent moisture absorption and oxidative degradation. These measures effectively maintained the integrity and consistency of the extract, minimizing any potential variability due to external factors such as seasonality, sampling location or environmental conditions.

The qualitative and quantitative composition of *Ulva pertusa* extract is detailed in our previous study [18].

### 4.2. Animals

Male Sprague-Dawley rats, aged 6 weeks and weighing between 200–250 g, were obtained from Envigo (Udine, Italy). Upon arrival, the rats were housed under standard laboratory conditions, which included a 12-h light/dark cycle and a controlled temperature of 22 ± 1 °C. They were provided with a standard diet and water and were observed in quarantine for 1 week before beginning the experiment. All animal procedures adhered to Italian legislation on the protection of animals used for scientific purposes (D.Lgs 2014/26), following EU regulations (EU Directive 2010/63) and ARRIVE guidelines. The animals selected for the study were randomly chosen from the pool of suitable specimens available at that time.

### 4.3. 5-FU Protocol

The 5-FU protocol was adapted from a previous study [47]. Rats in the 5-FU groups received intraperitoneal injections of 5-FU (fluorouracil 50 mg/mL) for 4 consecutive days at a dose of 15 mg/kg (human equivalent), followed by a reduced dose of 6 mg/kg administered on 4 alternate days. The final dose of 15 mg/kg was given on the 14th day of treatment. Sham rats, on the other hand, were treated with a vehicle (0.9% saline solution). Beginning on the 15th experimental day, oral administration of saline for Sham + vehicle and 5-FU + vehicle groups, or *Ulva pertusa* extract, was carried out once daily for 15 days. The dosage and route of administration for *Ulva pertusa* were selected based on prior studies in which 100 mg/kg proved the most effective dose in decreasing colonic pathological features on a dose-response pilot experiment, as previously reported [17,18]. Euthanasia of the animals was performed on the 30th day of the experimental protocol. At the end of the study, colons were surgically excised, embedded in paraffin, and prepared for histological analysis. Additionally, blood samples were collected for biochemical assessments.

### 4.4. Experimental Groups

The rats were allocated into the following experimental groups:

Group 1. Sham + vehicle: rats received intraperitoneal injections of the vehicle (0.9% saline solution), following the same schedule as the 5-FU protocol. Starting from the 15th experimental day, oral administration of the vehicle (saline) was conducted once daily for 15 consecutive days, continuing until the conclusion of the experiment on day 30 (N = 8).

Group 2. 5-FU + vehicle: rats were administered intraperitoneal injections of 5-FU at a dose of 15 mg/kg (human equivalent) for 4 consecutive days. The dosage was then lowered to 6 mg/kg, administered on 4 alternate days, with the final 15 mg/kg dose given on the 14th day. Starting on the 15th experimental day, oral treatments with the vehicle (0.9% saline solution) were provided once daily for 15 consecutive days, extending through day 30 of the experiment (N = 8).

Group 3. 5-FU + *Ulva pertusa*: rats were treated with intraperitoneal injections of 5-FU at 15 mg/kg (human equivalent) for 4 consecutive days, followed by a reduced dose of 6 mg/kg administered on 4 alternate days. The final dose of 15 mg/kg was given on the 14th day of treatment. Starting on the 15th experimental day, oral administration of *Ulva pertusa* was carried out once daily for 15 consecutive days until the end of the experiment on day 30 (N = 8).

### 4.5. Analysis of Body Weight, Food Intake, and Diarrhea Index

The body weight and food intake of each experimental group were measured and recorded weekly until the experiment concluded, using an electronic balance [48]. The severity of diarrhea was assessed using the following scale: 0 for normal (normal or no stool); 1 for slight (slightly wet and soft stool); 2 for moderate (wet, unformed stool with moderate blood); and 3 for severe (watery stool with significant rectal bleeding). This scale was used to calculate the diarrhea index for all experimental groups [49].

### 4.6. Von Frey and Light/Dark Examinations

Plantar pain sensitivity was assessed using calibrated von Frey filaments, which detect mechanical stimulus-response as an indicator of hyperalgesia induced by 5-FU administration [50]. The mechanical threshold (expressed in grams) corresponding to the pressure that elicited a behavioral reaction (paw withdrawal) was automatically recorded by an electronic device. Each stimulation was applied 3 times, and the average value was calculated as the mechanical threshold for each rat. Before measuring the mechanical paw withdrawal thresholds (PWT, expressed in grams), rats were acclimated to the behavioral chambers for 15 min. Mechanical thresholds were then assessed using an electronic Von Frey test (dynamic plantar aesthesiometer, model 37450; Ugo Basile, Italy) with a preset cutoff. Mechano-allodynia was identified by a significant (*p* < 0.05) reduction in the mean absolute PWT (g). Pain was evaluated at 3 time points: at the start of the experiment (day 0), after completion of the 5-FU protocol (day 15), and at the end of the experiment (day 30).

Photophobia, a symptom often associated with 5-FU-induced migraines, was quantified using a light/dark box [50]. The black and central gray chambers had their clear lids covered with heavy black construction paper to maintain low light levels (inside ≤ 5 lx), while the white chamber with clear lids served as the light portion (inside ≥ 635 lx). On the test day, rats were placed in the central chamber for a 1-min acclimation period before the guillotine-style doors were opened, granting access to the entire apparatus. The time spent in the light chamber and the total number of photobeam breaks during a 5-min test session were recorded at the beginning of the experiment (day 0), after completing the 5-FU protocol (day 15), and at the end of the study (day 30).

### 4.7. Histological Analysis

To evaluate morphological changes in the colon, we conducted histological analysis following the previously described method [51]. Immediately after sacrificing the animals, colon tissues were fixed in a 10% (weight/volume, *w*/*v*) PBS-buffered formaldehyde solution at 25 °C for 24 h. The tissues were then dehydrated through a graded alcohol series and subsequently embedded in paraffin (Bio-Optica, Milan, Italy). The samples were sectioned at 7 μm thickness using a microtome. Hematoxylin and eosin (H&E) staining (Bio-Optica, Milan, Italy) was performed to assess morphological changes, such as neutrophilic infiltration, edema formation, and alterations in colonic architecture. The histological analysis utilized the following criteria: score 0 for no morphological damage; score 1 for focal epithelial edema and necrosis; score 2 for diffuse inflammation and necrosis of the villous area; score 3 for neutrophilic infiltration in the submucosa; score 4 for necrosis with neutrophilic infiltration; and score 5 for extensive neutrophilic infiltration and bleeding. All stained sections were examined and analyzed in a blinded manner with a Nikon Eclipse Ci-L microscope, and the results were presented at 20× magnification (50 µm scale bar).

### 4.8. MPO Assay

Myeloperoxidase (MPO) activity, commonly used as a quantitative marker for neutrophil infiltration, was assessed in accordance with the protocol established in previous studies [51]. MPO, an enzyme abundantly present in neutrophils, catalyzes the production of reactive oxygen species during inflammatory responses, making its activity an important indicator of neutrophil accumulation in tissues. The assay was performed by measuring the change in absorbance spectrophotometrically at a wavelength of 650 nm, which corresponds to the reaction product of the MPO-catalyzed conversion of peroxide substrates. MPO activity in the tissue samples was expressed in units per gram of wet tissue, providing a standardized measure that allows for direct comparison between samples. This method ensures precise quantification of neutrophil infiltration, allowing for assessment of the inflammatory response within the examined tissue samples.

### 4.9. Immunohistochemistry

Immunohistochemical localization was performed as described in earlier studies [52]. Colon tissues were immediately fixed in a 10% (*w*/*v*) PBS-buffered formaldehyde solution at 25 °C for 24 h. Following dehydration through a series of increasing concentrations of alcohol and xylene, the tissues were embedded in paraffin (Bio-Optica, Milan, Italy), sectioned into 7 µm slices using a microtome, and then deparaffinized. Endogenous peroxidase activity was blocked using 0.3% (*v*/*v*) hydrogen peroxide in 60% (*v*/*v*) methanol for 30 min. The slides were permeabilized with 0.1% (*w*/*v*) Triton X-100 in PBS for 20 min. To reduce non-specific binding, the sections were incubated with 2% (*v*/*v*) normal goat serum in PBS for 20 min. Avidin and biotin binding sites were blocked through sequential incubation with avidin and biotin (Vector Laboratories, Burlingame, CA, USA) for 15 min each.

The tissue slices were then incubated overnight at room temperature with one of the following primary antibodies: anti-iNOS (BD Biosciences #610432, 1:100 in PBS, *v*/*v*), anti-COX-2 (Santa Cruz Biotechnology sc-376861, 1:100 in PBS, *v*/*v*), or anti-Nitrotyrosine (Millipore, Burlington, MA, USA, #06-284, 1:100 in PBS, *v*/*v*). After overnight incubation, the slides were washed with PBS and then incubated with a secondary antibody (Santa Cruz Biotechnology, Dallas, TX, USA) for 1 h. The reaction was visualized using a chromogenic substrate (brown DAB) and counterstained with Nuclear Fast Red. To confirm the specificity of antibody binding, some sections were incubated with only the primary or secondary antibody, and no positive staining was observed in these controls. All stained sections were examined and analyzed in a blinded manner. For immunohistochemical analysis, images were captured at 20× (50 µm scale bar) and 40× (20 µm scale bar) magnification.

### 4.10. Malondialdehyde (MDA) Assay

The level of MDA, widely recognized as a reliable biomarker for lipid peroxidation and oxidative stress, was measured in colon tissues following the protocol established in previous studies [18]. MDA is one of the final products formed by the degradation of polyunsaturated fatty acids and serves as an important indicator of oxidative damage within cell membranes. To assess MDA levels, colon tissue samples were processed and analyzed using a colorimetric assay, allowing for sensitive quantification. The results provide insight into the extent of lipid peroxidation, reflecting the oxidative status within the tissue and the impact of any oxidative stressors.

### 4.11. Hematocrit

After the rats were anesthetized, approximately 1 mL of blood was collected using a venous trocar catheter designed for animal-specific blood collection with a vacuum tube. The counts of red blood cells, white blood cells, and platelets were determined using an automated cell counter “automated hematology analyzer” (HecoVet C, SEAC, Florence, Italy). The quantities of red blood cells, white blood cells, and platelets were measured both after completing the 5-FU treatment protocol (day 15) and at the end of the experiment (day 30).

### 4.12. Assessment of Liver and Kidney Function

Blood was collected through cardiac puncture, and the serum was used to assess levels of aspartate aminotransferase (AST) (#ab263883), alanine aminotransferase (ALT) (#ab234579), urea (#MBS2600001), creatinine (#E02C0629), and uric acid (#ab65344) [47]. These analyses were conducted using commercial kits, with each measurement performed according to the manufacturer’s instructions.

ELISA kits were used to measure the levels of glutathione (GSH) (#MBS265966), catalase (CAT) (#MBS726781), superoxide dismutase (SOD) 1 (#MBS036924), and vitamin C (#MBS721134) in the serum of rats, and COX-2 (#MBS266603), iNOS (#MBS263618), and ROS (#MBS2802061) were evaluated in colon tissues. All the procedures were performed following the methods described previously [53] and according to the manufacturer’s protocols.

### 4.13. Statistical Analysis

Experimental data are presented as the mean ± standard deviation (SD) of N observations, where N represents the number of animals studied. Data were analyzed using one-way and two-way ANOVA, followed by a Bonferroni correction for multiple comparisons. A *p*-value of less than 0.05 was considered statistically significant.

## 5. Conclusions

For the first time, our research highlights the potential of *Ulva pertusa* as a promising adjuvant in chemotherapy. The findings reveal that *Ulva pertusa* not only modulates the inflammatory response but also enhances the body’s antioxidant defense mechanisms, offering protection against oxidative stress and cellular damage. This dual action could reduce the risk of chronic inflammatory disorders and improve overall resilience to oxidative stress. These promising attributes suggest that *Ulva pertusa* may have broader applications, both as a dietary supplement and a therapeutic adjuvant, in conditions characterized by oxidative stress and inflammation. Its potential role in disease prevention and treatment paves the way for future research, unlocking new possibilities for integrating this natural resource into modern healthcare.

## Data Availability

All data in this study are included in this published article.
